# Effects of stress on pain in females using a mobile health app in the Russia-Ukraine conflict

**DOI:** 10.1038/s44184-023-00043-w

**Published:** 2024-01-10

**Authors:** Aliaksandr Kazlou, Kateryna Bornukova, Aidan Wickham, Vladimir Slaykovskiy, Kimberly Peven, Anna Klepchukova, Sonia Ponzo, Sarah Garfinkel

**Affiliations:** 1 Flo Health UK Limited, London, United Kingdom; 2 BEROC Economic Research Center, Minsk, Belarus; 3 Universidad Carlos III de Madrid, Department of Economics, Madrid, Spain; 4 University College London, Institute of Health Informatics, London, United Kingdom; 5 University College London, Institute of Cognitive Neuroscience, London, United Kingdom

**Keywords:** Human behaviour, Signs and symptoms, Psychology

## Abstract

The chronic and acute effects of stress can have divergent effects on health; long-term effects are associated with detrimental physical and mental health sequelae, while acute effects may be advantageous in the short-term. Stress-induced analgesia, the attenuation of pain perception due to stress, is a well-known phenomenon that has yet to be systematically investigated under ecological conditions. Using Flo, a women’s health and wellbeing app and menstrual cycle tracker, with a world-wide monthly active usership of more than 57 million, women in Ukraine were monitored for their reporting of stress, pain and affective symptoms before, and immediately after, the onset of the Russian-Ukrainian conflict. To avoid potential selection (attrition) or collider bias, we rely on a sample of 87,315 users who were actively logging multiple symptoms before and after the start of the war. We found an inverse relationship between stress and pain, whereby higher reports of stress predicted lower rates of pain. Stress did not influence any other physiological symptoms with a similar magnitude, nor did any other symptom have a similar effect on pain. This relationship generally decreased in magnitude in countries neighbouring and surrounding Ukraine, with Ukraine serving as the epicentre. These findings help characterise the relationship between stress and health in a real-world setting.

## Introduction

Cognitive, perceptual and sensory processing are influenced by the dynamic nature of bodily states^1^. Stress states are associated with a cascade of autonomic and neuroendocrine responses which can modify subsequent processing. The phenomenon of ‘stress’ is a multifaceted concept, with no agreed definition or consensus in the literature. A classical view of stress refers to homoeostatic challenges that require an adaptive response^2^. Stress can either facilitate or impair a range of cognitive functions, depending on the nature of the stress and the specific type of processing^3^. As such, acute stress can have adaptive effects; for example, memory for information encoded during stressful experiences can be facilitated^4^, especially if this information is relevant to the stressor^5–7^. While the impacts of stress on cognition have dominated the literature, with a particular focus on memory, perceptual and sensory processing are also affected. Acute stress is associated with decreased pain sensitivity, a phenomenon known as ‘stress-induced analgesia’ (SIA)^8^. Pain can capture attention^9^ and cognitive and affective processing bi-directionally interact with the experience of pain^9,10^. A reduction in pain processing during times of stress could aid the prioritising of resources for adaptive responding^11^, yet may pose severe health consequences in the long term.

The phenomenon of SIA has been observed in wounded soldiers in World War II, who reported a reduced level of pain compared to those suffering from comparable injuries that occurred in non-threatening environments^12^. These initial observations highlighted the role of context in shaping pain perception, with a particular focus on the role of stress. Informed by ecological experiences, SIA has been subsequently studied almost exclusively in the laboratory. Following exposure to stressful stimuli, laboratory studies routinely demonstrate a pain suppression response. This finding is well established and well replicated, with a succession of lab-based studies detailing underlying anatomical, neurochemical and molecular mechanisms subserving this effect^8,13–15^.

While the initial observations of SIA were informed by armed conflict^12^, these were anecdotal in nature and inspired a wealth of systematic follow-up studies in the laboratory^15–17^. Further study and evaluation of SIA within ecological settings is required, although the pursuit of this has been hampered by the practical difficulties of systematically measuring stress and pain responses during extremely stressful situations, such as armed conflict. The health consequences of armed conflicts are devastating both in terms of direct (e.g. war casualties) and indirect (e.g. disruption of long-term healthcare services) costs^18^. War can severely affect the health of those involved, with long-term effects on overall physical and mental health^19^. Research conducted in countries either experiencing civil wars (e.g. Sri Lanka) or wars against other countries (e.g. Balkans) highlighted the wide presence of psychiatric disorders, such as depression, anxiety and post-traumatic stress disorder (PTSD), in civilians^20^. The mental health impacts on women specifically have also been well documented, e.g. Afghanistan^21,22^ and the Balkans^23^, where a higher vulnerability to stress was driven by a range of factors, including a sudden requirement to increase their level of societal and familial responsibility within an extreme stress context^24^.

At the time of writing, the current Russia-Ukraine conflict (starting 24th of February 2022) is ongoing, marked by an increasing death toll with profound damage to Ukraine and its infrastructure^25^. Millions have been forced to leave their homes to seek refuge in neighbouring countries, and all men of age have been conscripted. The magnitude of health consequences is emerging, including in vulnerable groups such as mothers and newborns^26^. It is estimated that mental health sequelae will be long-lasting, building upon the aftermath of the COVID-19 pandemic^27^. Emerging research conducted six months after the war started found increased levels of anxiety, depression, and stress in a Ukrainian cohort with women found to be more vulnerable than men^28^. Exposure to war-related trauma and stressful events was predictive of anxiety, depression, and acute stress levels^28^.

With the advent of new techniques for real-time data collection via mobile phones and wearable devices, ecological studies of SIA are possible in extreme contexts such as war. Specifically, applications that track users’ mood and physical symptoms over time can provide us with a temporally relevant representation of individuals’ psychological and physiological states, and their dynamic relationship. A specific kind of popular and widely used mobile apps that track symptoms over time are menstruation trackers^29^. Users of menstruation-tracking apps often log symptoms throughout their menstrual cycle, allowing apps to provide them with personalised predictions of future symptom patterns and estimates of menstrual phases. Flo^30^ (by Flo Health UK Limited) is a women’s and people who menstruate’s health and wellbeing mobile app. Flo supports the logging of menstruation, ovulation, and symptoms such as pain, stress, anxiety and other bodily and emotion-based metrics. Flo has the biggest worldwide audience among health and fitness apps, exceeding 57 million monthly active users. As such, it is uniquely positioned to provide valuable insights into physiological and psychological patterns and phenomena at scale. With real-time logging and location-specific data, symptoms such as pain and stress can be monitored in real-time as highly stressful events unfold.

This paper aimed to investigate a real-world instance of SIA for women in Ukraine by characterising the relationship between self-reported stress and pain in an extreme danger context. Specifically, we explored logging patterns in a cohort of users of the period tracker Flo residing in Ukraine before and during the conflict. We hypothesised that an inverse relationship would exist, whereby individuals reporting stress would be less likely to report pain at the start of the conflict, consistent with the acute effects of SIA. We expected these effects to diminish with time after the start of the invasion as Ukrainians became accustomed to the context of ongoing war. We hypothesised that the acute effects of SIA would be observable in surrounding/neighbouring European countries, but not to the magnitude that would be observed in Ukraine. While the long-term consequences of stress on physical and mental health can be highly detrimental and pervasive, this transient acute effect would be consistent with adaptive mechanisms of short-term survival.

## Results

### Symptom descriptives before and after the start of the war

**Fig. 1:**
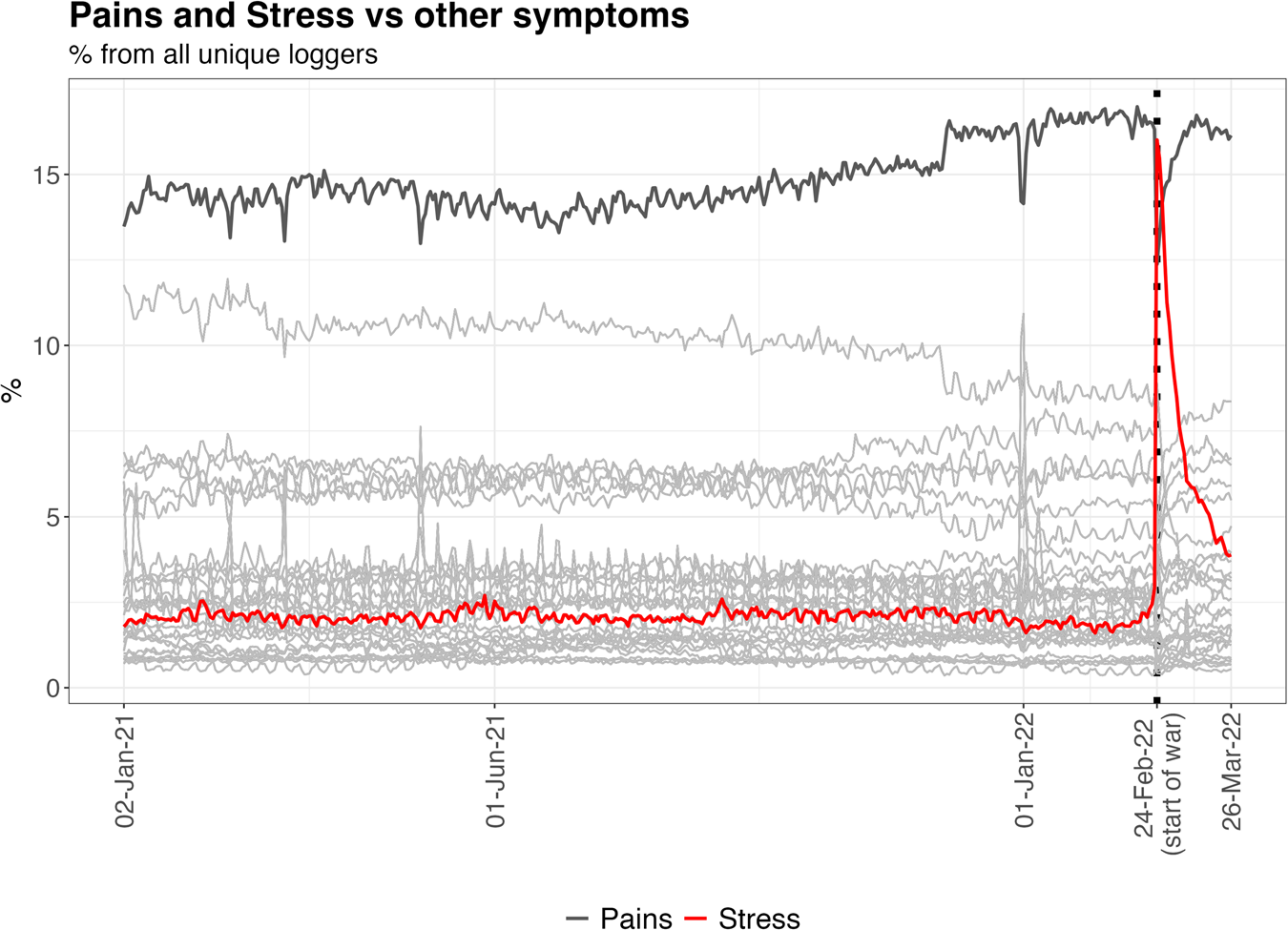
Symptoms logged by users of the Flo app (full sample) over a year for illustration purposes. Symptom prevalence is reported relative to the total number of users who log at least one symptom each day. We highlight stress and pains (rather than all other symptoms, presented in light grey) to detail their inverse relationship.

Descriptive statistics were obtained for the prevalence of logging patterns 30 days before and 30 days after the war started. Most notably, the largest change in prevalence was observed for stress, increasing from 3% of all unique users who logged any symptom on February 23rd to 16% on February 24th. Several pain-related symptoms (headache, backache, and abdominal pain) decreased in prevalence from before to after the war started. When averaged together, the prevalence of these grouped ‘pains’ (headache, backache, abdominal pain, cramps, and perineum pain) decreased after the war started - from 16% to 12%. As shown in Fig. 1, there is an inverse relationship between logged stress and ‘pains’ at the start of the war; the prevalence of logged stress was consistent within the sample for over a year before the war started.

### The effects of stress and other symptoms on pain

**Fig. 2:**
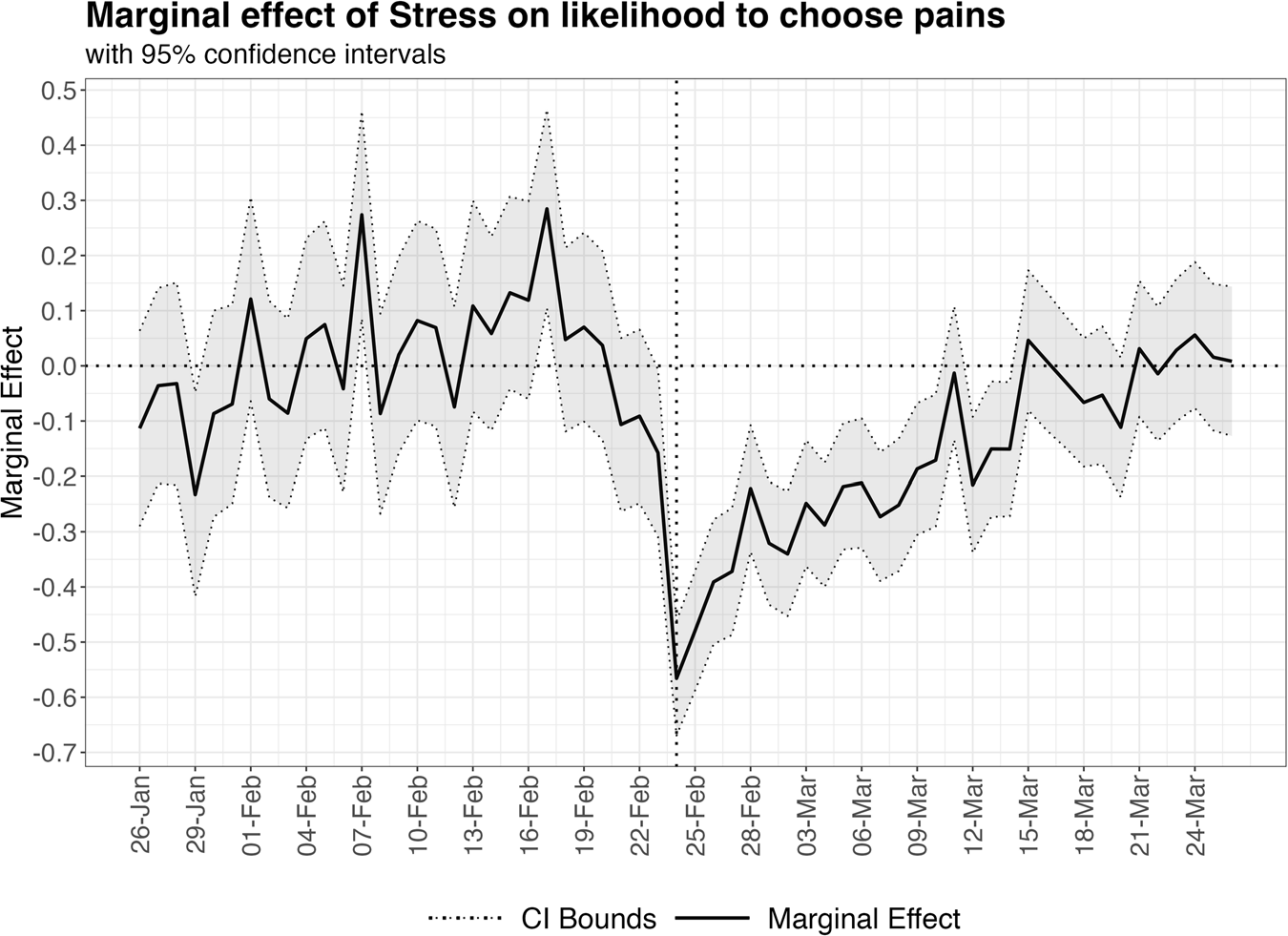
Effect of stress on pain over time. Marginal effects and confidence intervals for the effect of logging stress on the likelihood to log pains.

Figure 2 presents the marginal effects of stress on pain over time, indicating a substantial decline in the effect of stress that gradually returns to pre-war levels in the following weeks. The day before the war, the marginal effect of stress on pain was −0.161 (0.077), however, the following day when the war started it decreased to -0.572 (0.053) and remained low and significant for 15 consecutive days until March 11, 2022. The marginal effects and standard errors of each logit model on each date shown in Fig. 1 are presented in Supplementary Table 1.

**Fig. 3:**
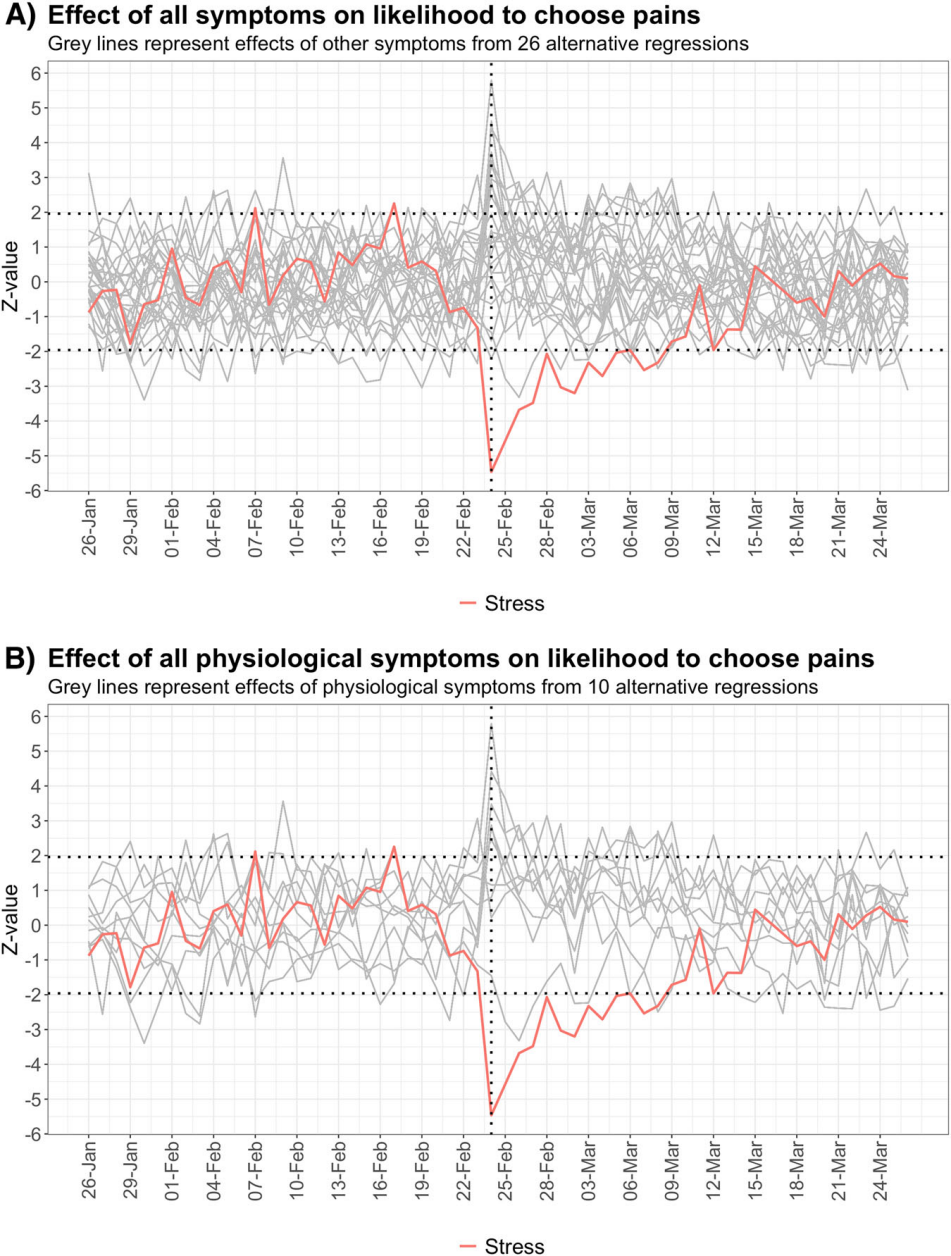
Time fixed effect interaction with alternative independent variables to stress. **A** Z-statistic for the time fixed effect interaction with every other independent symptom variable from 26 alternative regressions **B** Z-statistic for the time fixed effect interaction with other physiological independent symptom variables from 10 alternative regressions.

To demonstrate the specificity of the relationship between stress and pain, we conducted 26 alternative regressions by substituting stress in the time interaction term with other control variables (symptoms that could be logged by users). Figure 3A, B display z-statistics for the interaction effects between the control variables and the time fixed effects. The results indicate that the relationship with pain changed for several symptoms after the onset of the conflict, such as an upward dynamic for acne from 2022-02-24 to 2022-02-26. However, stress was found to have the most profound and long-lasting effect on reducing the likelihood of logging pain-related symptoms after 2022-02-24.

**Fig. 4:**
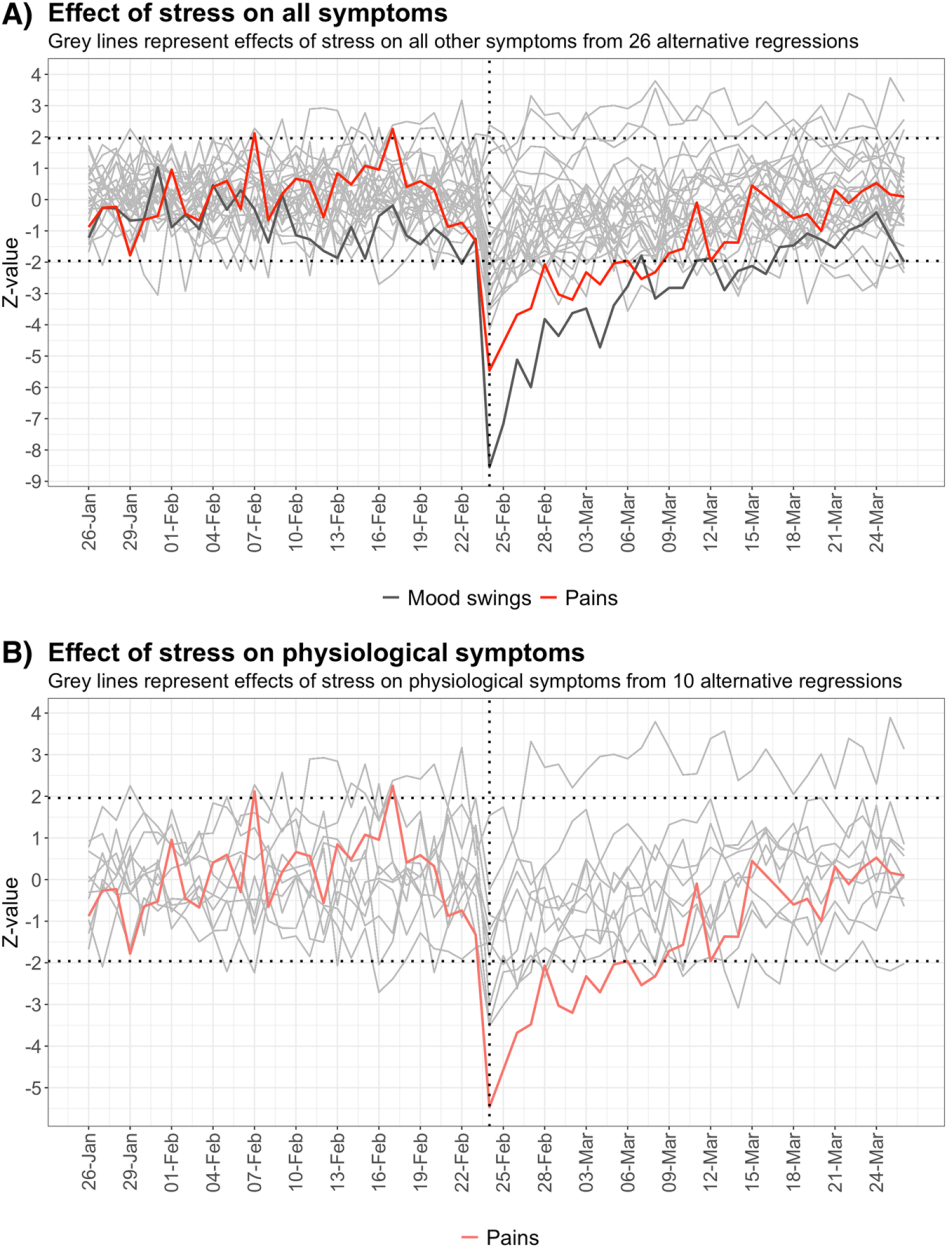
Stress and time fixed effect interaction on alternative dependent variables to pain symptoms. **A** Z-statistic for the stress and time fixed effect interaction on every other dependent symptom variable from 26 alternative regressions. **B** Z-statistic for the stress and time fixed effect interaction on other physiological dependent symptom variables from 10 alternative regressions.

Furthermore, we substituted pain symptoms with other symptoms as the dependent variable of our logit model to demonstrate how they are affected by stress, shown in Fig. 4A and Fig. 4B. We find a similar attenuating effect in other significantly affected symptoms including mood-symptoms ‘mood swings’ and ‘feeling calm’. However, pain remains the most affected physiological symptom and the second most affected symptom overall after ‘mood swings’.

### Geographical representation

**Fig. 5:**
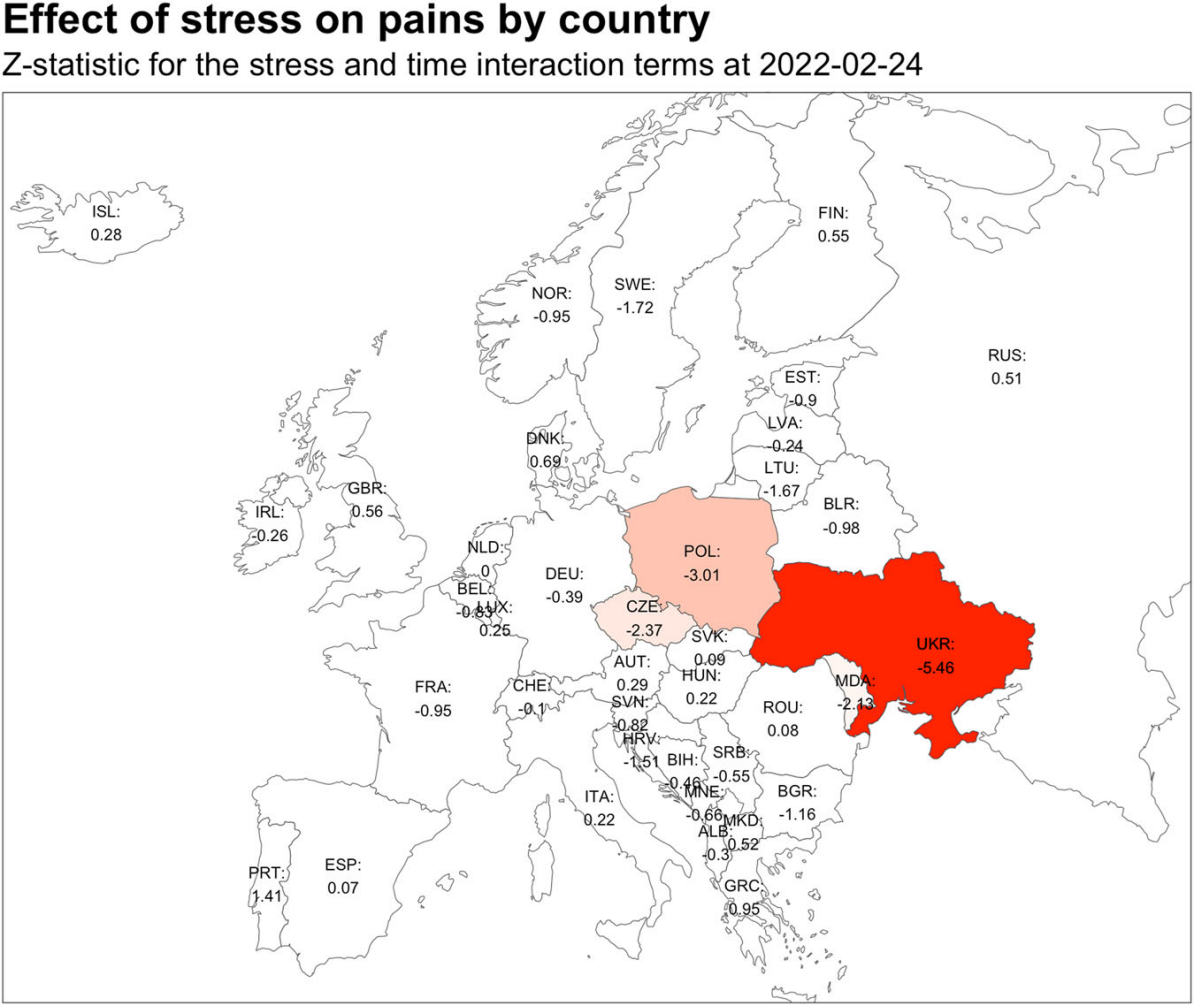
Effect of stress on pain by country. Z-statistic for the stress and time fixed effect interaction for 38 European countries at 2022-02-24.

Our investigation into the regional specificity impact of logged stress on pain looked at 38 in the European countries surrounding Ukraine on the day the war started: Ukraine, Russia, Germany Sweden, France, Hungary, Great Britain, Poland, Belarus, SwitzerlandItaly, Spain Romania, Lithuania, Serbia, Finland, Belgium, Netherlands, Norway, Austria, Greece Portugal, Latvia, Estonia, Croatia, Ireland, Slovakia, Czechia, North Macedonia, Denmark, Slovenia, Albania Moldova, Bulgaria, Iceland, Luxembourg, Bosnia and Herzegovina, and Montenegro. The results are represented graphically in Fig. 5 and Supplementary Table 2. Our findings indicate that the largest effect, presented as the Z-statistic for the stress and time fixed effect interaction term, was observed in Ukraine (-5.46), which was anticipated. We also found that other countries close to Ukraine were significantly impacted by stress: Poland (-3.01), Czechia (-2.37), and Moldova (-2.13).

## Discussion

The current study aimed to characterise the relationship between self-reported stress and pain in the context of the ongoing war. We found that users of the Flo app residing in Ukraine when the Russia-Ukraine conflict started exhibited logging patterns consistent with the phenomenon of stress-induced analgesia (SIA). Specifically, logging stress significantly decreased the likelihood of logging pains in this cohort.

After the war started, the likelihood of logging symptoms in the pain category significantly decreased for users logging stress. To the best of our knowledge, this is the first documented real-life example of SIA in a large cohort in a heightened stress context, such as during the onset of an invasion. Notably, the effect of stress on pain symptoms was specific to pain and did not extend to other physiological symptoms, except for mood-related symptoms such as mood swings or feeling calm, which are understandably impacted by stress. Furthermore, no other symptom showed a similar reduction in response to stress. In essence, stress had a greater impact on reducing pain symptoms than on any other physiological symptom, and pain symptoms were reduced the most by stress.

Despite the immediate evolutionary advantage that a phenomenon like SIA provides, extreme traumatic situations, such as war, have proven detrimental effects on individuals’ long-term health. Exposure to severe trauma leads to worse health outcomes and disability in women veterans^31^ as well as in civilians^32^. Similarly, more significant long-term mental health distress is associated with reports of traumatic experiences in war refugees^33^. In addition to individual health consequences, long-term health repercussions of war and trauma represent a significant economic burden for healthcare systems^34^. Research has shown increased levels of stress in Ukrainian populations due to the recent invasion, with women being particularly vulnerable to mental health issues^28^. With Ukraine and neighbouring countries already suffering from significant losses, staff shortages and cuts to their healthcare systems due to the ongoing conflict^35,36^, caring for these special populations could be particularly difficult. Further to shortages, healthcare providers may lack the specific expertise to address war-related trauma and effects of forced displacement, with the most vulnerable populations (e.g. children) being exposed to increased risk of developing long-term mental health consequences^37^. Solutions should employ approaches that address resilience, are multi-level, trauma-informed and group-specific to maximise outcomes^37–39^. Chronic pain and PTSD frequently co-occur; a high prevalence of individuals with PTSD also experience chronic pain. While prevalence rate varies depending on the study^40,41^, of particular note is work detailing that in 129 consecutive veterans with PTSD, 80% were also found to be suffering from co-occurring chronic pain^42^. Similar patterns have been observed in female veterans, where pain related symptoms significantly predicted PTSD symptom cluster scores^43^. While the relationship between PTSD and chronic pain is well established, less is known about the time-course of acute stress and pain, and whether the magnitude of this effect may serve as a potential risk factor for the later development of comorbid chronic pain and PTSD.

Within our data, the relationship between stress and pain steadily decreased over time from the day the war started, providing important data suggesting the stress-analgesic effect is short-lived. Understanding the time-course of naturally occuring SIA can complement previous work based in the laboratory which invariably had a restricted time window under which such effects could be studied^34^. Similarly, the effects of stress per se at the group level also returned to baseline within the 30-days following the start of the war. Together, these results are consistent with high levels of stress promoting an analgesic effect, an effect that declined as acute stress levels decreased. Further, the relationship between stress and pains was greatest in individuals based in Ukraine, demonstrating this pain induced analgesic effect was greatest in individuals most impacted by the conflict.

Further research providing concurrent measurement of autonomic and neural patterns could provide insight into underlying mechanisms of stress induced analgesia. Stress responses are associated with changes in cardiovascular signals^44^, and previous lab-based work demonstrates that discrete cardiac signals (at systole, when the brain processes the degree of cardiovascular arousal), are associated with an attenuated nociceptive reflex response^45^, reduced pain evoked potentials^46^ and changes in amygdala, anterior insula and pons activation underlying pain processing^47^. These findings suggest a dynamic relationship between heightened sympathetic arousal and attenuated pain processing, compatible with the phenomenon of stress-induced analgesia. Future work probing autonomic reactivity using wearable technology to combine with app based responses have the potential to scale-up psychophysiology stress research in real-world contexts.

A limitation of the current paper is that we consider symptom logging to be a proxy for true psychological and physiological patterns. Self-tracked data with optional entry may be less reliable than empirical protocols requiring regular data entry. In terms of tracking behaviours, users have different propensity to log symptoms based on their perceived importance, thus making it difficult to distinguish between tracking behaviours and the actual presence and/or absence of symptoms. Finally, it should be noted that we only considered location settings the first time we downloaded the data - we did not look at location dynamically over the course of the conflict.

Given that we focused on the logged symptoms only, the issue of sample selection may arise. The sample selection could potentially be subject to attrition bias (as we are missing symptom data from the subsample of the population that did not log their symptoms during the war) and collider bias (i.e. users become more likely to log one symptom only, and this symptom is stress). Indeed, the total number of logged symptoms and the amount of unique loggers decreased in the month after the start of the war compared to the pre-war period. However, our focus is on the dynamic relationship between stress and reported pain within users who were active both before and after the war started, and this specific relationship is less likely to be affected by sample selection. Our focus is on users who logged at least two symptoms on a given day which makes it less likely to be affected by collider bias.

In conclusion, the current study utilises app-based technology to investigate the phenomenon of stress-induced analgesia in the context of an armed conflict. In line with the acute and adaptive mechanisms of short-term survival, we found that users in Ukraine who reported stress were less likely to report pain following the onset of the war. Despite the potential short-term adaptive response of stress on pain, stress is associated with a future cost; heightened stress has established detrimental effects on long-term mental and physical health.

## Methods

### Participants and dataset

Our analysis included Flo app users whose smartphone location settings were set to Ukraine during their last login as of 2022-04-06 and who were active on the app between 2022-01-25 and 2022-03-26 (i.e. 30 days before and 30 days after the start of the war). The total number of Ukrainian users registered on the Flo app is 3,732,111. Of those, 201,572 users logged symptoms 30 days before the start of the war and 196,311 users 30 days after the start of the war. The total number of symptoms logged is 1,844,546 before the start of the war and 1,807,209 after the start of the war (see Supplementary Table 3a).

To avoid bias (e.g. non-random absence of individual data-points after the start of the war), we first restricted our main analysis to a subsample of users who logged at least two symptoms throughout the selected time-window We further restricted our sample to users who logged at least two symptoms on any given day. For these 87,315 users, the total number of symptoms logged is 1,234,133 before the start of the war and 1,176,613 after the start of the war (see Supplementary Table 3b). Age is optional data for Flo users to provide and can be subject to error; when calculating the mean age and age-range of our sample we excluded 5,487 users with either missing or implausible self-reported age. The mean self-reported age of the sample was 21.1 years (±6.5 years) with an age-range of 11-39 years (81,828 users, after excluding outliers and missing age data).

Written consent was waived as all participants provided informed electronic consent for their data to be used for research purposes when agreeing to the usage terms of the Flo app; the study protocol was approved by an independent ethics review board (Western Copernicus Group Independent Review Board - WCG IRB).

### Materials and measures

#### Flo app

**Fig. 6:**
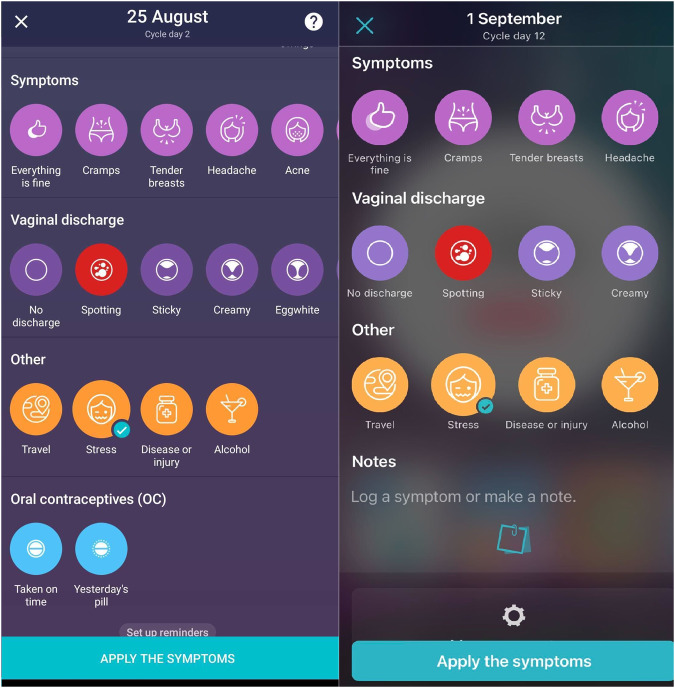
Screenshots of the Flo app symptoms panel. Users can log each symptom once per day at any time throughout the day. Symptoms are organised by category, including: Sex and sex drive (e.g. High sex drive, Unprotected sex), Mood (e.g. Calm, Sad, Happy), Symptoms (e.g. Cramps, Headache, Tender breasts), Vaginal discharge (e.g. Sticky, Spotting, Creamy), Other (e.g. Travel, Stress). Left: screenshot from an Android phone; Right: from iOS.

Users of the Flo app can track their menstruation, ovulation, pregnancy, and symptoms throughout their menstrual cycle and are offered AI-based menstruation and ovulation predictions. The Flo app also offers an extensive educational library containing a wide range of topics related to women and people who menstruate’s health and wellbeing, as well as personalised content based on the symptoms the user has logged. The Flo app is available for free to download on both iOS and android operating systems. Users can pay for a premium subscription giving them access to more features and educational content, but both free and premium users have access to logging their symptoms through the ‘symptoms panel’ shown in Fig. 6. Users can log their physical symptoms (e.g. cramps, headache, bloating), mood (e.g. happy, anxious), sex and sex drive (e.g. whether unprotected sex occurred), vaginal discharge (e.g. creamy, watery) as well as the occurrence of stress, travel or alcohol intake (see Fig. 6). Each symptom can be logged once daily and different symptoms can be added throughout the day (e.g. users can log “Stress” at 10 am and subsequently add “Bloating” at 5 pm, but cannot add “Stress” again until the following day). The strength of symptoms is not tracked.

Flo provides users with evidence-based and expert-reviewed content throughout their cycle via both the in-app library and through health assistants (chatbots). Health assistants also allow users to check their symptoms against a list of symptoms provided in the medical guidelines from accredited institutes and organisations. Flo users can also ask questions and provide answers to other users worldwide anonymously, thus reducing the perceived stigma surrounding topics such as sexual life and menstrual health^48–50^.

The set of analysed symptoms consists of physiological symptoms, mood-related symptoms, and stress. The list of physiological symptoms includes cramps, headache, abdominal pain, perineum pain, backache, tender breasts, swelling, fatigue, acne, bloating, disease or injury, nausea, cravings, diarrhoea, and insomnia. The list of mood-related symptoms includes calm, frisky, depressed, irritated, mood swings, happy, apathetic, feeling guilty, energetic, sad, obsessive thoughts, anxious, confused, and very self-critical. The set of analysed symptoms has been consistently reported by users with minimal impact from any changes made to the app.

#### Modelling the effect of stress on pains

To examine the analgesic effect of stress, we developed a model using a restricted sample of users who logged at least two symptoms on a given day. The dependent variable was assigned a value of 1 if a pain symptom was logged and 0 otherwise. The independent variables were binary indicators for stress and the 26 other available symptoms (controls) that could be logged. We also controlled for time fixed effects and the interaction between stress and time fixed effects to account for time-variant dynamics. The marginal effect after the war started might serve as a proxy to strength of stress. We estimate the following logit model in Eq. 1:

1 $${Prob}({pai}{n}_{{it}})=f\left({\beta }_{0}+{\beta }_{1}{stres}{s}_{{it}}+\mathop{\sum }\limits_{j=1}^{T}{\beta }_{2j}\,{dat}{e}_{j}{{\cdot }}{stres}{s}_{{it}}+{\beta }_{3}{dat}{e}_{t}+\mathop{\sum }\limits_{k=1}^{26}{{\beta }^{k}}_{4}{contro}{{l}^{k}}_{{it}}+{\epsilon }_{{it}}\right)$$

where *i* denotes individual from 1 to N; *t* is the time subscript from date 1 to T; *date*_*j*_ is a time fixed effect, a dummy variable which takes the value of 1 at time *j*, and 0 otherwise; k is the index for 1 to 26 additional symptoms used as controls. This formulation of the estimated model allows for time-variant marginal effect of stress. Reported standard errors are heteroskedasticity-robust standard errors (HC1).

Our operationalization ensures that we included only active users who had sufficient time to open the app and log at least two symptoms i.e. a user recorded stress as one of their symptoms but not any pain-related symptoms, the user had the option to log pain but decided to log something else instead. This reasoning extends to all other possible symptom combinations. Furthermore, to mitigate the attrition bias associated with non-random absence of individual data points after the start of the conflict, we restricted our analysis to individuals who were active at least one day 30 days before and 30 days after the onset of conflict.

In order to assess the regional specificity of Ukraine, we examined the impact of stress on pains in users in European countries that neighbour and surround Ukraine. For each country, we analysed the Z-statistic of the stress and time fixed effect interaction term from our logit model at the start of the war on 2022-02-24.

### Reporting summary

Further information on research design is available in the Nature Research Reporting Summary linked to this article.

### Supplementary information

Reporting summary

Supplementary information

## Data Availability

The data used for the present study, including detailed tables of regression statistics, can be made available through reasonable requests to the corresponding author due to data sharing restrictions.

## Code availability

The code used for the present study, including detailed tables of regression statistics, can be made available through reasonable requests to the corresponding author.
