# Supplementary material for: Effects of stress on pain in females using a mobile health app in the Russia-Ukraine conflict
Source: Npj Ment Health Res. 2024 Jan 10;3:2. doi: 10.1038/s44184-023-00043-w (PMC10956037; doi:10.1038/s44184-023-00043-w)
Supplement: Supplementary file 1 — Reporting summary [file 44184_2023_43_MOESM1_ESM.pdf]

Table 1: Marginal effect of stress on pain over time

	Date	Estimate
1	2022-01-26	-0.113 (0.090)
2	2022-01-27	-0.036 (0.090)
3	2022-01-28	-0.032 (0.094)
4	2022-01-29	-0.233 (0.094)*
5	2022-01-30	-0.087 (0.095)
6	2022-01-31	-0.069 (0.092)
7	2022-02-01	0.121 (0.094)
8	2022-02-02	-0.060 (0.091)
9	2022-02-03	-0.086 (0.087)
10	2022-02-04	0.049 (0.093)
11	2022-02-05	0.075 (0.096)
12	2022-02-06	-0.042 (0.095)
13	2022-02-07	0.274 (0.096)**
14	2022-02-08	-0.087 (0.092)
15	2022-02-09	0.020 (0.091)
16	2022-02-10	0.082 (0.092)
17	2022-02-11	0.069 (0.091)
18	2022-02-12	-0.075 (0.093)
19	2022-02-13	0.109 (0.098)
20	2022-02-14	0.059 (0.090)
21	2022-02-15	0.132 (0.089)
22	2022-02-16	0.119 (0.091)
23	2022-02-17	0.285 (0.092)**
24	2022-02-18	0.048 (0.085)
25	2022-02-19	0.070 (0.087)
26	2022-02-20	0.037 (0.087)
27	2022-02-21	-0.106 (0.080)
28	2022-02-22	-0.091 (0.080)
29	2022-02-23	-0.158 (0.077)*
30	2022-02-24	-0.566 (0.053)***
31	2022-02-25	-0.480 (0.055)***
32	2022-02-26	-0.391 (0.058)***
33	2022-02-27	-0.372 (0.059)***

34	2022-02-28	-0.222 (0.058)***
35	2022-03-01	-0.321 (0.057)***
36	2022-03-02	-0.340 (0.058)***
37	2022-03-03	-0.249 (0.058)***
38	2022-03-04	-0.288 (0.057)***
39	2022-03-05	-0.218 (0.058)***
40	2022-03-06	-0.212 (0.060)***
41	2022-03-07	-0.273 (0.059)***
42	2022-03-08	-0.252 (0.061)***
43	2022-03-09	-0.187 (0.061)**
44	2022-03-10	-0.171 (0.061)**
45	2022-03-11	-0.013 (0.062)
46	2022-03-12	-0.215 (0.063)**
47	2022-03-13	-0.151 (0.062)*
48	2022-03-14	-0.151 (0.062)*
49	2022-03-15	0.046 (0.065)
50	2022-03-16	0.009 (0.064)
51	2022-03-17	-0.029 (0.061)
52	2022-03-18	-0.066 (0.059)
53	2022-03-19	-0.053 (0.064)
54	2022-03-20	-0.112 (0.064)
55	2022-03-21	0.031 (0.063)
56	2022-03-22	-0.014 (0.062)
57	2022-03-23	0.028 (0.066)
58	2022-03-24	0.056 (0.068)
59	2022-03-25	0.016 (0.068)
60	2022-03-26	0.008 (0.069)
*p<0.05; **p<0.01; ***p<0.001		

Table 2: Output for the stress and time interaction terms at 2022-02-24 from 38 regressions for European countries

	Country	N	Estimate	Statistic
1	RUS	85848	0.057 (0.111)	0.51
2	DEU	86595	-0.036 (0.092)	-0.393
3	SWE	26665	-0.278 (0.162)	-1.72
4	FRA	86934	-0.096 (0.102)	-0.945
5	HUN	5938	0.077 (0.343)	0.225
6	GBR	88979	0.056 (0.099)	0.564
7	POL	87496	-0.23 (0.076)* *	-3.012
8	BLR	47376	-0.132 (0.135)	-0.976
9	CHE	27770	-0.018 (0.181)	-0.1
10	ITA	89778	0.018 (0.081)	0.216
11	ESP	87136	0.007 (0.095)	0.074
12	ROU	17170	0.016 (0.196)	0.081
13	LTU	9238	-0.45 (0.269)	-1.672
14	SRB	5972	-0.2 (0.362)	-0.553
15	FIN	22394	0.09 (0.164)	0.547
16	BEL	24103	-0.146 (0.177)	-0.828
17	NLD	36147	0.001 (0.144)	0.004
18	NOR	19584	-0.201 (0.211)	-0.952
19	AUT	29550	0.046 (0.16)	0.287
20	GRC	7677	0.316 (0.333)	0.949
21	PRT	28458	0.24 (0.17)	1.409
22	LVA	6786	-0.075 (0.316)	-0.238
23	EST	6007	-0.315 (0.351)	-0.898
24	HRV	5806	-0.536 (0.356)	-1.506
25	IRL	31234	-0.046 (0.178)	-0.259
26	SVK	2896	0.043 (0.46)	0.094
27	MKD	9483	0.349 (0.671)	0.521
28	CZE	2354	-0.602 (0.253)*	-2.374
29	DNK	9341	0.241 (0.347)	0.695
30	SVN	6422	-0.279 (0.342)	-0.817
31	ALB	3621	-0.149 (0.503)	-0.296
32	MDA	4943	-1.058 (0.497)*	-2.128
33	BGR	3994	-0.488 (0.423)	-1.156
34	ISL	2236	0.185 (0.652)	0.284
35	LUX	2490	0.151 (0.606)	0.249
36	BIH	2137	-0.267 (0.585)	-0.457
37	MNE	765	-0.842 (1.272)	-0.662
38	UKR	87315	-0.563 (0.103)* * *	-5.464
*p<0.05; **p<0.01; ***p<0.001				

Table 3a: Descriptive statistics (full sample)

	N before war	N after war	Symptom	% before war	% after war
1	121937	95984	Calm	9.2	7.8
2	118578	112816	Cramps	8.9	9.2
3	85158	73616	Tender_breasts	6.4	6
4	78728	61483	Fatigue	5.9	5
5	76366	70267	Mood_swings	5.8	5.7
6	70090	59226	Headache	5.3	4.8
7	68266	64950	Acne	5.1	5.3
8	64477	55309	Everything_is_fine	4.9	4.5
9	63454	55702	Backache	4.8	4.5
10	45379	38158	Bloating	3.4	3.1
11	39074	33354	Cravings	2.9	2.7
12	38476	39447	Irritated	2.9	3.2
13	37475	25099	Happy	2.8	2
14	35931	40537	Sad	2.7	3.3
15	32368	11803	Disease_or_injury	2.4	1
16	31480	35329	Depressed	2.4	2.9
17	30974	25322	Nausea	2.3	2.1
18	28516	46087	Anxious	2.1	3.7
19	28317	18644	Energetic	2.1	1.5
20	25265	15044	Alcohol	1.9	1.2
21	24482	82664	Stress	1.8	6.7
22	21349	24350	Obsessive_thoughts	1.6	2
23	18994	16324	Insomnia	1.4	1.3
24	18037	12251	Frisky	1.4	1
25	17583	15700	Diarrhea	1.3	1.3
26	17441	17587	Apathetic	1.3	1.4
27	16731	15145	Abdominal_pain	1.3	1.2
28	16033	18499	Confused	1.2	1.5
29	11082	10499	Constipation	0.8	0.9
30	10662	8430	Swelling	0.8	0.7
31	10253	9471	Perineum_pain	0.8	0.8
32	9488	7658	Very_self_critical	0.7	0.6
33	8960	8451	Feeling_guilty	0.7	0.7
34	5977	6730	Travel	0.5	0.5

February 24, 2022 is excluded as day 0 (start of the war)

Table 3b: Descriptive statistics (restricted sample)

	N before war	N after war	Symptom	% before war	% after war
1	118060	92617	Calm	9.6	8.1
2	98440	93065	Cramps	8	8.2
3	75924	59637	Fatigue	6.2	5.2
4	72892	67323	Mood_swings	5.9	5.9
5	70695	61266	Tender_breasts	5.7	5.4
6	65698	55784	Headache	5.3	4.9
7	64973	61684	Acne	5.3	5.4
8	60491	53047	Backache	4.9	4.7
9	60461	51667	Everything_is_fine	4.9	4.5
10	41933	35288	Bloating	3.4	3.1
11	36738	24586	Happy	3	2.2
12	36505	37547	Irritated	3	3.3
13	35634	30321	Cravings	2.9	2.7
14	35575	40213	Sad	2.9	3.5
15	30057	34184	Depressed	2.4	3
16	29318	24006	Nausea	2.4	2.1
17	27379	44664	Anxious	2.2	3.9
18	27119	17752	Energetic	2.2	1.6
19	26177	10661	Disease_or_injury	2.1	0.9
20	22830	64239	Stress	1.8	5.6
21	21439	13062	Alcohol	1.7	1.1
22	21276	24292	Obsessive_thoughts	1.7	2.1
23	17749	15306	Insomnia	1.4	1.3
24	17278	17436	Apathetic	1.4	1.5
25	17106	11614	Frisky	1.4	1
26	16350	14837	Abdominal_pain	1.3	1.3
27	15957	14361	Diarrhea	1.3	1.3
28	15204	17860	Confused	1.2	1.6
29	10580	8359	Swelling	0.9	0.7
30	10509	9882	Constipation	0.9	0.9
31	9888	9140	Perineum_pain	0.8	0.8
32	9444	7629	Very_self_critical	0.8	0.7
33	8939	8433	Feeling_guilty	0.7	0.7
34	5515	6386	Travel	0.4	0.6

February 24, 2022 is excluded as day 0 (start of the war)
