# Supplementary material for: Effects of stress on pain in females using a mobile health app in the Russia-Ukraine conflict
Source: Npj Ment Health Res. 2024 Jan 10;3:2. doi: 10.1038/s44184-023-00043-w (PMC10956037; doi:10.1038/s44184-023-00043-w)
Supplement: Supplementary file 2 — Supplementary information [file 44184_2023_43_MOESM2_ESM.pdf]

nature portfolio

Corresponding author(s): Aidan Wickham

Last updated by author(s): Mar 15, 2023

Reporting Summary

Nature Portfolio wishes to improve the reproducibility of the work that we publish. This form provides structure for consistency and transparency in reporting. For further information on Nature Portfolio policies, see our Editorial Policies and the Editorial Policy Checklist.

Statistics

For all statistical analyses, confirm that the following items are present in the figure legend, table legend, main text, or Methods section.

n/a	Confirmed	
☐	☒	The exact sample size (*n*) for each experimental group/condition, given as a discrete number and unit of measurement
☐	☒	A statement on whether measurements were taken from distinct samples or whether the same sample was measured repeatedly
☐	☒	The statistical test(s) used AND whether they are one- or two-sided<br>*Only common tests should be described solely by name; describe more complex techniques in the Methods section.*
☐	☒	A description of all covariates tested
☐	☒	A description of any assumptions or corrections, such as tests of normality and adjustment for multiple comparisons
☐	☒	A full description of the statistical parameters including central tendency (e.g. means) or other basic estimates (e.g. regression coefficient) AND variation (e.g. standard deviation) or associated estimates of uncertainty (e.g. confidence intervals)
☐	☒	For null hypothesis testing, the test statistic (e.g. *F*, *t*, *r*) with confidence intervals, effect sizes, degrees of freedom and *P* value noted<br>*Give P values as exact values whenever suitable.*
☒	☐	For Bayesian analysis, information on the choice of priors and Markov chain Monte Carlo settings
☒	☐	For hierarchical and complex designs, identification of the appropriate level for tests and full reporting of outcomes
☒	☐	Estimates of effect sizes (e.g. Cohen's *d*, Pearson's *r*), indicating how they were calculated

Our web collection on statistics for biologists contains articles on many of the points above.

Software and code

Policy information about availability of computer code

Data collection	Flo database queried using Trio 931 built into custom Python scripts
Data analysis	Python 3.8.9 with libraries: Prestodb, Trino, Pandas, Numpy Matplotlib, Seaborn, Scikit, Statsmodels.

For manuscripts utilizing custom algorithms or software that are central to the research but not yet described in published literature, software must be made available to editors and reviewers. We strongly encourage code deposition in a community repository (e.g. GitHub). See the Nature Portfolio guidelines for submitting code & software for further information.

Data

Policy information about availability of data

All manuscripts must include a data availability statement. This statement should provide the following information, where applicable:

- Accession codes, unique identifiers, or web links for publicly available datasets
- A description of any restrictions on data availability
- For clinical datasets or third party data, please ensure that the statement adheres to our policy

Data is available upon request due to data sharing restrictions.

Human research participants

Policy information about studies involving human research participants and Sex and Gender in Research.

Reporting on sex and gender	Flo is a health app used by women and people who menstruate across the globe. We do not ask our users information regarding their gender identification.
Population characteristics	See 'Behavioural & social sciences'. Age range collected was 13-100; note: age is provided by users and sometimes inaccurate (i.e. users 100 years old). Age is not used in the analysis.
Recruitment	Users were selected based on their geolocation status being in Ukraine on 2022-04-06.
Ethics oversight	Independent Ethical Review Board (WCG IRB)

Note that full information on the approval of the study protocol must also be provided in the manuscript.

Field-specific reporting

Please select the one below that is the best fit for your research. If you are not sure, read the appropriate sections before making your selection.

☐ Life sciences ☒ Behavioural & social sciences ☐ Ecological, evolutionary & environmental sciences

For a reference copy of the document with all sections, see nature.com/documents/nr-reporting-summary-flat.pdf

Behavioural & social sciences study design

All studies must disclose on these points even when the disclosure is negative.

Study description	Using Flo, a women's health app with a world-wide monthly active usership of more than 48 million, women in Ukraine were monitored for their reporting of symptoms before, and immediately after, the onset of the Russian-Ukrainian conflict. The data analysed is categorical/dichotomous symptom data - i.e. each symptom is logged as a binary value for its presence or not on a particular date; quantitative severity of a symptom is not possible in the app.
Research sample	Our analysis included Flo app users whose smartphone location settings were set to Ukraine during their last login as of 2022-04-06 and who were active on the app between 2022-01-25 and 2022-03-26. The rationale was to include users who were still present in Ukraine a month after the war started, and still actively using the Flo app (so as to not mistake their inactivity in the app as the absence of a logged symptom). This is a retrospective cohort study; the sample of users was chosen based on their presence on Ukraine on 2022-04-06, and the data analysed has already been logged in the app.
Sampling strategy	The total number of Ukrainian users registered on the Flo app is 3,732,111. Of those, 201,572 users logged symptoms 30 days before the war and 196,311 users 30 days after the war. The total number of symptoms logged is 1,844,546 before the war and 1,807,209 after the war. To avoid bias (e.g. non-random absence of individual data-points after the start of the war), we further restricted our sample to 87,315 users who logged at least two symptoms on any given day.
Data collection	Our analysis included users whose smartphone location settings were set to Ukraine during their last login as of 2022-04-06 and who were active on the app between 2022-01-25 and 2022-03-26.
Timing	Data was collected from Flo app data logged between 2022-01-25 and 2022-03-26.
Data exclusions	Ukrainian users who did not log as least once in the month before the start of the war and at least once in the month after the war had started.
Non-participation	No participants dropped out, but were only excluded based on the reasons given in the sampling strategy
Randomization	N/A

Reporting for specific materials, systems and methods

We require information from authors about some types of materials, experimental systems and methods used in many studies. Here, indicate whether each material, system or method listed is relevant to your study. If you are not sure if a list item applies to your research, read the appropriate section before selecting a response.

Materials & experimental systems

n/a	Involved in the study
☒	☐ Antibodies
☒	☐ Eukaryotic cell lines
☒	☐ Palaeontology and archaeology
☒	☐ Animals and other organisms
☒	☐ Clinical data
☒	☐ Dual use research of concern

Methods

n/a	Involved in the study
☒	☐ ChIP-seq
☒	☐ Flow cytometry
☒	☐ MRI-based neuroimaging
